# Unusual Manifestations of Kawasaki Disease in the COVID Era: A Case Series and Review of the Literature

**DOI:** 10.7759/cureus.51104

**Published:** 2023-12-26

**Authors:** Maria Kostara, Anastasios Serbis, Maria Pavlou, Eleni Kotanidou, Sofia Tsabouri, Antonios Vlahos, Alexandros Makis, Ekaterini Siomou

**Affiliations:** 1 Department of Pediatrics, University Hospital of Ioannina, Ioannina, GRC; 2 Second Department of Pediatrics, School of Medicine, Faculty of Health Sciences, Aristotle University of Thessaloniki, AHEPA University General Hospital, Thessaloniki, GRC

**Keywords:** kawasaki disease (kd), mis-c, atypical presentation, pediatric case, medium vessel vasculitis

## Abstract

Kawasaki disease (KD) is an acute medium-vessel vasculitis, mainly affecting infants older than six months and children under five years. It predisposes to the development of coronary artery aneurysms and constitutes the leading cause of acquired heart disease in children. Its diagnosis is based on clinical criteria, namely, fever lasting for ≥ five days together with at least four of the five principal clinical features of the disease. Occasionally, children with KD present with fever, but they fulfill only some of the five principal criteria, and this is described as incomplete KD. Furthermore, “atypical” KD is a term that is usually used for cases that appear with rather unusual clinical manifestations, which complicate clinical judgment and may delay diagnosis and treatment. In this case series, we present four cases of KD with rather unusual clinical features: a five-year-old boy with lobar pneumonia, a six-year-old girl with orange-brown chromonychia appearing on the 10^th^ day of the disease, a 2.5-month-old infant with prolonged fever and urinary tract infection, and an 18-month-old infant with refractory KD and high suspicion of multisystem inflammatory syndrome in children (MIS-C). A literature review on the unusual manifestations of atypical KD was performed to identify clinical findings that must alert the clinician to consider this clinical entity.

## Introduction

Kawasaki disease (KD) is an acute medium vessel vasculitis of unknown etiology, which affects mainly infants older than six months and children up to five years [1]. It predisposes to the development of coronary artery aneurysms, and it constitutes the leading cause of acquired heart disease in children. It was first described in Japan in 1970, by Dr. Tomisaku Kawasaki and has a worldwide distribution. Its prevalence is higher in children of Asian origin and in boys [2,3]. There are no diagnostic tests available to confirm the disease. Therefore, its diagnosis is based on clinical criteria, namely, fever lasting for five days or more together with at least four of its five principal clinical features [4]. These include cervical lymphadenopathy (usually unilateral), bilateral non-exudative conjunctivitis, oral mucous membrane changes such as erythema/cracking of lips, strawberry tongue, and/or erythema of oral and pharyngeal mucosa, changes in the extremities, including erythema and edema of the hands and feet, and skin polymorphous rash [5]. In some cases, children with KD present with a fever lasting five days or more, but they do not fulfill four of the five clinical criteria and this is described as incomplete KD. In addition, children can appear with the so-called “atypical KD,” i.e., with rather unusual clinical manifestations that may mislead or complicate clinical judgment and may delay disease diagnosis and appropriate treatment [6]. In this case series, we present four cases of KD with rather unusual clinical features: a five-year-old boy with lobar pneumonia, a six-year-old girl with orange-brown chromonychia appearing on the 10th day of the disease, a 2.5-month-old infant with prolonged fever and urinary tract infection, and an 18-month-old girl with high suspicion of multisystem inflammatory syndrome in children (MIS-C) due to recent SARS-CoV-2 infection. Based on these cases, we reviewed the literature on the unusual findings of atypical KD and described clinical findings that should alert the clinician in considering the diagnosis of KD even if unusual clinical findings are present.

## Case presentation

In this article, we report four cases of children with atypical KD. These children were hospitalized in the Pediatric Department of the University Hospital of Ioannina, Greece, between July 2022 and April 2023. To obtain the relevant literature on atypical manifestations of KD, a search on PubMed/MEDLINE database was conducted for papers in English in children aged 0-18 years, between January 1, 1980, and May 31, 2023, using the following keywords: “atypical Kawasaki disease,” “Kawasaki unusual manifestations,” “Kawasaki rare manifestations,” “Kawasaki unusual signs.” Clinical case reports, case series, observational studies, and systematic reviews were included in the initial evaluation. Duplicates were excluded by title and relevance was evaluated according to title and abstract, where available. Full-text articles for all relevant studies were retrieved and reviewed. A manual search of the references from the retrieved articles led to additional relevant papers that were also included.

Case presentation 1

A five-year-old boy was transferred to the pediatric department of our hospital from a secondary regional hospital. Five days prior, he was hospitalized there with a fever (maximum temperature (Tmax): 39.5°C), which was persistent and responding poorly to acetaminophen or ibuprofen, painful bilateral cervical lymphadenopathy, and a maculopapular rash, with a progressively worsening general condition. Upon arrival at our hospital, his temperature was 36.7°C, his oxygen saturation was 95% on room air, his heart rate was 130/minute, and his blood pressure on his right upper limb was 99/59 mmHg. On examination, the boy was irritable and pale. His tonsils were enlarged, had cracked lips and painful bilateral cervical lymphadenopathy. Moreover, his upper eyelids were swollen, heart sounds were normal, whereas fine crackles were heard over the right lower lobe. The abdomen was soft without tenderness and hepatomegaly without any splenomegaly. There was no skin rash. Laboratory testing revealed the following: a total white blood cell (WBC) count of 17,930/mL with neutrophilic predominance (78%), platelet count of 408,000/μL, hemoglobin of 8.9 g/dL, erythrocyte sedimentation rate (ESR) of 94 mm/hour (first hour), and C-reactive protein (CRP) of 320 mg/L. Urinalysis was normal. Serological tests for Epstein-Barr virus (EBV) and cytomegalovirus (CMV) were negative. The results of the lumbar puncture showed pleocytosis. FilmArray Respiratory Panel (BioFire Diagnostics, Salt Lake City, UT) (multiplex polymerase chain reaction) testing for pathogens of the upper respiratory tract was positive for human rhinovirus and enterovirus. Chest radiograph showed right lower lobe consolidation with air bronchogram and bilateral pleural effusions. Ultrasound of the lower thorax and abdomen showed bilateral pleural effusion of about 20 mL on each side. Since his initial hospital admission, he had been started on broad-spectrum antibiotics (cefotaxime 150 mg/kg/day and clindamycin 40 mg/kg/day). Because of the identified pneumonia, clindamycin was discontinued, and vancomycin 60 mg/kg/day was added to the therapeutic regimen. On the second day of his admission, the patient was still febrile, he developed a strawberry tongue, and he underwent echocardiography, which revealed diffuse dilatation of the coronary arteries (CA), especially on the main left. In view of the echocardiography results, the persistent fever for over five days, the clinical findings (cervical lymphadenitis, cracked lips, rash), and the elevated inflammatory markers, the child was diagnosed with incomplete KD (since he only fulfilled three out of five criteria), while at the same time, he had the atypical manifestation of bacterial pneumonia. He was started on intravenous immunoglobulin (IVIG) (2 g/kg) on the second day of his hospitalization and on the sixth day of his disease. The fever subsided within four hours after the IVIG therapy. Due to glucose-6-phosphate dehydrogenase (G6PD) deficiency, clopidogrel rather than aspirin was started at an antiplatelet dose (1 mg/kg), 48 hours after the fever subsided. On the fourth day of his hospitalization, he presented a desquamation of the hands and the feet while the other clinical and laboratory parameters gradually improved. On the 9th day of his hospitalization, echocardiography showed a mild ectasia of the main left CA without aneurysm. The child was discharged on the 11th day with a maximum platelet count of 892,000/μL. Follow-up echocardiography 15 and 30 days after discharge was normal.

Case presentation 2

**Figure 1:**
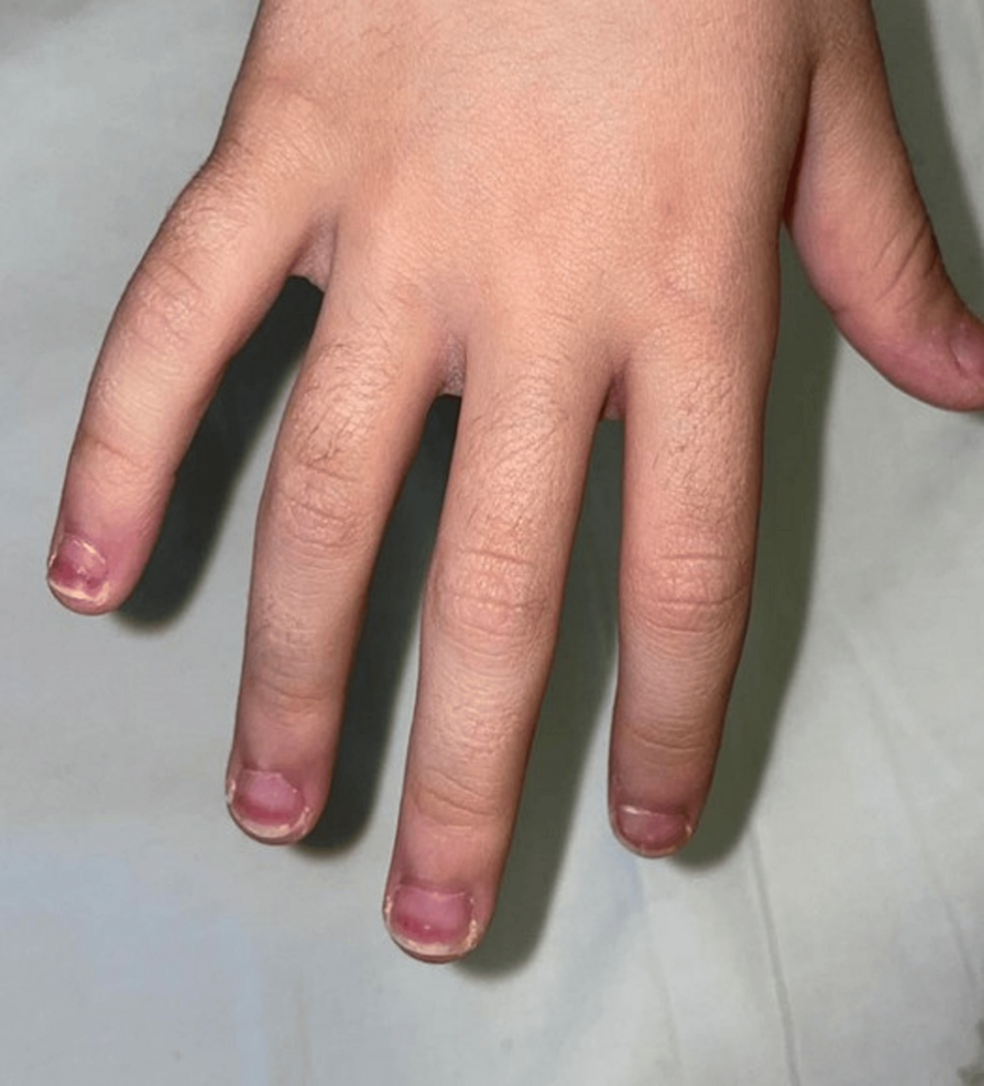
Orange-brown chromonychia in the second patient

A six-year-old girl was transferred to the pediatric department of our hospital. Six days prior, she had been admitted to a secondary regional hospital because of a fever for two days (reported Tmax: 40°C), painful unilateral cervical lymphadenopathy, diarrhea, and inflamed tonsils. The fever lasted for eight days in total. Despite not being feverish anymore, she was transferred to the tertiary hospital because of the clinical and laboratory findings raising the possibility of KD. Upon admission, her temperature was 36.7°C, her oxygen saturation was 97% on room air, her heart rate was 120/minute, and her blood pressure on her right upper limb was 115/85 mmHg. On examination, she presented conjunctivitis of the right eye, tonsillitis, cracked lips, and unilateral cervical lymphadenopathy. The examination of the cardiovascular and respiratory systems was normal. Moreover, she had a soft abdomen and no skin rash. The laboratory findings revealed the following: a total WBC of 12,520/μL with a predominance of lymphocytes (48.4%), platelet count of 634,000/μL (maximum count during her hospitalization), hemoglobin of 12.8 g/dL, ESR of 83 mm/hour, and CRP of 49 mg/L. Urinalysis was normal. Virology workup (EBV, CMV) was negative. Both throat swab and blood cultures were sterile. Chest radiography and ultrasound of the abdomen did not show any abnormal findings. In addition, she underwent an echocardiography on the first day of her hospitalization, which revealed normal ejection fraction and normal coronary arteries. Regarding treatment, she was receiving broad-spectrum antibiotics (cefotaxime 150 mg/kg and clindamycin 40 mg/kg) since her initial admission. On the second day after her admission (10th day of disease), she was afebrile, but irritable, and developed a desquamation of the hands and orange-red discoloration of the distal half of her fingernails (Figure 1). According to the above-mentioned findings, which are supportive of KD disease, she was treated as an atypical KD case. Treatment with IVIG (2 g/kg) and aspirin at an antiplatelet dose (3 mg/kg) started immediately. Twenty-four hours after treatment, both her clinical condition and the laboratory tests improved. With regard to the discoloration of the fingernails, they gradually became darker, orange-brown. On the 8th day of her hospitalization (16th day of disease), a repeat echocardiogram was normal. The child was discharged on the 9th day after admission. Follow-up echocardiography two months after discharge revealed no abnormal findings.

Case presentation 3

A 2.5-month-old female infant was admitted to the pediatric department of our hospital because of a fever for 18 hours (Tmax: 39°C), and reduced feeding and urination. On admission, her temperature was 36°C, her oxygen saturation was 98% on room air, and her heart rate was 135/minute. At clinical examination, she was pale, sleepy, and with decreased muscle tone. The anterior fontanelle was open flat. She was grunting, but lung auscultation had no abnormal sounds. No other abnormal physical findings were observed, except for a systolic murmur (1/6) on the fourth intercostal space. Initial laboratory results were as follows: a total WBC of 11,150/μL with lymphocyte predominance (68.3%), platelet count of 140,000/μL, hemoglobin of 9.1 g/dL, ESR of 47 mm/hour (first hour), CRP of 30 mg/L, serum alanine aminotransferase (ALT) of 304 IU/L (normal range (NR): 9-50), and aspartate aminotransferase (AST) of 342 IU/L (NR: 20-67). Urinalysis revealed WBC of 3-5/hpf and 2+ leucocyte esterase and *Klebsiella pneumoniae* 105 cfu/mL was isolated in the urine culture. Lumbar puncture and chest radiography were normal. Regarding her antibiotic treatment, she has been on wide-spectrum antibiotics (cefotaxime 150 mg/kg and ampicillin 200 mg/kg) since her admission. Amikacin 15 mg/kg/24 hours was added while ampicillin was discontinued after the results of the urine culture according to the antibiogram (resistant to ampicillin and cefotaxime, sensitive to amikacin). On the 4th day after her admission, the fever persisted, she was more irritable, and a maculopapular rash on her body and limbs was detected together with cracked lips and swollen hands and feet. Moreover, her laboratory tests deteriorated: WBC of 22,230/μL, with neutrophil predominance (65%), hemoglobin of 6.8 g/dL (she underwent a red blood cell transfusion), platelets of 544,000/μl, and CRP of 89 mg/L. Due to the clinical findings and the laboratory results, on the 5th day after admission (6th day of fever), she was treated as an atypical KD with IVIG (2 g/kg) and aspirin at an antiplatelet dose (5 mg/kg). After the IVIG treatment, the fever subsided. During her hospitalization, two echocardiograms (2nd and 4th day after admission) showed no CA dilation. In addition, the abdomen ultrasonography was normal. On the 12th day after admission, desquamation of the hands was noted. The infant was discharged on the 16th day of hospitalization. Her clinical condition improved as well as her laboratory findings (maximum platelet count during hospitalization: 978,000/μL). After the discharge, she continued receiving aspirin (5 mg/kg) for three weeks as the echocardiogram was normal.

Case presentation 4

An 18-month-old girl was admitted because of a six-day history of fever (Tmax: 40°C) and poor appetite. On admission, she was afebrile, her oxygen saturation was 97% on room air, her heart rate was 148/minute, and her blood pressure on her right upper limb was 102/56 mmHg. Regarding her clinical examination, she had bilateral non-purulent conjunctivitis, cracked lips, strawberry tongue, bilateral cervical lymphadenopathy, and a diffuse maculopapular rash at the trunk and limbs. Furthermore, her hands and feet were swollen. Laboratory tests upon admission showed the following: a total WBC of 20,660/μL with neutrophilic predominance (61.9%), platelet count of 393,000/μL, hemoglobin of 10.6 g/dL, ESR of 72 mm/hour (first hour), CRP of 117 mg/L, and normal cardiac enzymes. Her urinalysis revealed sterile pyuria. Chest radiography showed peri-bronchial thickening, ultrasound of the abdomen, and enlarged mesenteric lymph nodes, while her echocardiogram showed normal CA. Since her admission, she was treated with a broad-spectrum antibiotic (cefotaxime 150 mg/kg). Due to the fulfillment of the criteria of KD disease, she was treated with IVIG (2 g/kg) and oral aspirin (at first at an anti-inflammatory dose of 40 mg/kg in four divided doses). After the IVIG infusion, the fever subsided for some hours but reappeared 24 hours later. Therefore, she received a second 2 g/kg IVIG course, along with methylprednisolone 2 mg/kg in two divided doses with a tentative diagnosis of refractory KD disease or a high suspicion of MIS-C syndrome due to a history of recent (two months before) COVID infection and positive IgG antibodies for SARS-CoV-2: 538.7 AU/mL (normal <50 AU/mL). In addition, she fulfilled the clinical criteria for MIS-C, including age (0-19 years), fever for more than three days, rash, elevated D-dimers (as evidence of coagulopathy), elevated markers of inflammation, and no other obvious cause of inflammation [7]. Fever subsided some hours after the second course of IVIG and glucocorticoids initiation and on the 4th day after the admission, desquamation of the hands and feet was noted. The echocardiogram was repeated twice (on the 3rd and the 9th day after admission) with normal findings. Her laboratory findings as well as her clinical condition improved gradually. The maximum platelet count during her hospitalization was 544,000/μL. She was discharged on the 14th day of her hospitalization. She continued receiving aspirin at an antiplatelet dose (5 mg/kg) for two months after discharge.

## Discussion

**Figure 2:**
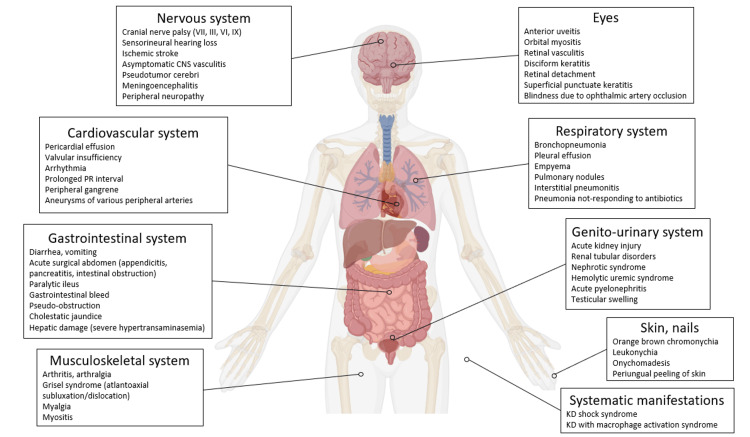
Atypical Kawasaki disease manifestations reported in the literature according to the systems involved KD: Kawasaki disease. The figure was designed by the authors using BioRender.

Although KD diagnosis is based on fever for at least five days together with ≥ four out of five clinical criteria, sometimes not all of these criteria are fulfilled at the same time, making the diagnosis rather challenging. In such cases, the term incomplete KD is used. Further, even if the term “atypical KD” has been used interchangeably with incomplete KD, it should be rather reserved for cases that present with one or more unusual signs or symptoms of KD from various systems. Several unusual signs and symptoms from various systems such as the central nervous system, the skin (other than typical KD rash), the kidneys, or the lungs have been described in the literature (Figure 2).

The presence of such manifestations can disorientate clinical judgment, leading to diagnostic confusion, and delayed diagnosis and treatment, thus increasing CA aneurysm risk. In this case series, we described four cases of children with atypical KD presentation and unusual clinical findings. In addition, an extensive literature review was conducted to identify KD cases with atypical manifestations that have been described before. An analysis of the literature revealed a male preponderance (789 males vs. 572 girls), while no significant association between atypical KD manifestations and age of presentation (infants vs. children), or specific system involved, was identified. Pulmonary involvement is not a usual presentation in KD. Khoury et al. described two infants with atypical KD who presented at first as pneumonia. In the first infant, pneumonia was due to *Streptococcus* group A, whereas the other one was infected both with *Streptococcus* and influenza type A virus. In both infants, fever subsided after IVIG treatment [8]. In another study conducted by Singh et al., pulmonary involvement was described in 1.83% of children with KD. Chest X-ray of these children revealed consolidation and their main clinical features were cough, fever, and respiratory distress. Most of the patients were treated with IVIG after 20 days of fever. This treatment delay increases the risk of CA aneurysms [9,10]. In our case, the five-year-old child with prolonged fever and chest X-ray consolidation was treated with IVIG on the 7th day of fever. Within hours of treatment initiation, the child became afebrile, his clinical condition improved considerably, and follow-up echocardiography was normal. Therefore, KD should be considered in children with prolonged fever and abnormal chest X-ray findings in whom the clinical condition does not improve on antibiotic treatment alone. Apart from the pulmonary involvement, nail lesions may also appear in children suffering from KD. Specifically, orange-brown chromonychia is a nail lesion that appears at the acute phase of KD [11]. In the study by Pal et al., patients with KD presented this nail lesion between the 5th and the 8th day after fever onset [12]. In another study by Mitsuishi et al., orange-brown chromonychia was the most common nail abnormality in patients with KD, even though such a clinical finding remains quite rare [13]. In our six-year-old patient, orange-brown chromonychia appeared on the 10th day of the disease and assisted in the correct diagnosis and treatment of her atypical KD. It is therefore important for the clinician to have a high index of suspicion when it comes to atypical KD cases and to be able to recognize various non-specific clinical presentations of the disease. As already mentioned, KD affects mainly children aged six months to five years of age. The incidence of KD in infants younger than three months of age is 1.6% [14]. KD diagnosis in infancy is more complicated as the presentation is usually incomplete [1]. Even in infants who present only with prolonged fever for more than five days and no other symptoms, KD should be considered in the differential diagnosis [15]. Our 3rd case, a 2.5-month-old infant, presented with prolonged fever and urinary tract infection due to *Klebsiella pneumoniae* and was finally diagnosed with atypical KD and treated accordingly. Sterile pyuria is a common finding in atypical KD, especially in infants younger than six months of age [16]. Nevertheless, in our case, pyuria was not sterile as *Klebsiella pneumoniae* (105 cfu/mL) was isolated from the catheterized urine culture. The current case is one of the very few described in the literature of infants < six months having urinary tract infections and atypical KD at the same time. Wu et al. described two children aged six and 19 months, respectively, who suffered from urinary tract infections and atypical KD [17]. These infants also presented dilation of the CA. In infants younger than six months with prolonged fever, elevated CRP, and pyuria, it is of great importance not to miss the diagnosis of atypical KD, even if other diagnoses could initially justify the clinical and laboratory findings. Our fourth patient presented with refractory KD, which required a second dose of IVIG for the fever and inflammation to subside. What made the diagnosis more difficult was his history of a recent SARS-CoV-2 infection and the positive IgG antibodies for the virus. MIS-C, a hyperinflammatory response to SARS-CoV-2 infection, can have very similar clinical manifestations with KD. The fact that most patients with MIS-C can also meet the criteria for KD makes initial diagnosis and treatment decisions quite challenging. What can help in the differential diagnosis is the age of the patient (KD is more frequent in younger patients), and the presence of CA aneurysms, which are more frequently observed in patients with KD, while MIS-C patients present more frequently with elevated cardiac enzymes, ventricular dysfunction, and hemodynamic instability. In addition, gastrointestinal symptoms, an increased coagulation state, elevated D-dimers, and ferritin, together with thrombocytopenia and lymphopenia, all point to MIS-C. On the contrary, increased WBCs with eosinophilia and high platelet numbers are more frequently associated with KD [18]. Our fourth patient fulfilled both KD and MIS-C criteria and presented with several clinical characteristics of both diseases. Since no cardiac or coronary involvement, that could help in the differential diagnosis, was identified, a definite diagnosis was not possible [19]. Therefore, the patient was treated with IVIG, glucocorticoids, and aspirin, covering both KD and MIS-C possible diagnoses, and responded well to the therapy without any long-term sequelae (Table 1).

**Table 1:** Studies reporting unusual/rare clinical manifestations of Kawasaki disease (atypical Kawasaki disease) KD: Kawasaki disease.

Clinical manifestations	Reference Νο.	Number of patients	Age, months and years	Male	Female
Bronchopneumonia	[8]	11	6 months-4 years	5 males	6 females
Empyema	[9]	2	15-28 months		2 females
Pleural effusion	[10]	1	3 years		Female
Pulmonary nodules	[20]	3	4-6 months	Males	
Interstitial pneumonitis	[21]	2	2-14 months		Females
Pneumonia not responding to antibiotic treatment	[22]	2	2-2.5 years	1 male	1 female
Orange-brown chromonychia	[11]	1	5 years		Female
Orange-brown chromonychia	[12]	29	Not reported	Not reported	Not reported
Orange-brown chromonychia	[13]	17	3 months-6 years	Not reported	Not reported
Leukonychia	[13]	7	3 months-6 years	Not reported	Not reported
Onychomadesis	[13]	6	3 months-6 years	Not reported	Not reported
Periungual peeling of skin	[13]	1	30 months		Female
Acute kidney injury	[23]	93	2 months-4^7/12 ^years	53 males	40 females
Renal tubular disorders	[24]	39	3 months–45 years	27 males	12 females
Acute pyelonephritis	[17]	2	6-18 months	Males	
Testicular swelling	[25]	2	19-59 months	Males	
Anterior uveitis	[26]	41	27-65 months	18 males	23 females
Orbital myositis	[27]	1	8 months	Male	
Retinal vasculitis	[28]	1	4 years	Male	
Disciform keratitis	[29]	1	11 years	Male	
Retinal detachment	[30]	1	11 years		Female
Superficial punctuate keratitis	[31]	4	5 months–9 years	Males	Females
Blindness due to ophthalmic artery occlusion	[32]	1	9 years		Females
Arthritis (usually of large joints)	[33]	8	2-10 years	5 males	3 females
Arthritis	[34]	151	2 months–13 years	89 males	62 females
Grisel syndrome (atlantoaxial subluxation/dislocation)	[35]	9	4-9 years	2 males	7 females
Myalgia	[36]	1	6 years		Female
Myositis	[36]	1	6 years		Female
Diarrhea, vomiting	[37]	106	6 months-5 years	63 males	43 females
Acute surgical abdomen	[38]	10	3 months-10 years	7 males	3 females
Acute surgical abdomen	[39]	13	4-7 years	Males	Females
Acute surgical abdomen	[40]	1	2 years	Male	
Acute surgical abdomen	[41]	2	9-12 years	Males	
Paralytic ileus	[42]	1	18 months	Male	
Gastrointestinal bleed	[43]	2	5-7 years	Males	
Pseudo-obstruction	[44]	49	-	Males	Females
Cholestatic jaundice	[45]	1	9 years		Female
Hepatic damage	[46]	1	1 year	Male	
Cranial nerve palsy (VII, III, VI, IX)	[47-49]	44	2 months-13 years	Males	Females
Sensorineural hearing loss	[50]	3	16 months-10.5 years	Males	
Ischemic stroke	[51]	1	15 months	Male	
Asymptomatic CNS vasculitis	[52]	6	1-6 years	1 male	6 females
Peripheral neuropathy	[53]	80	7 months-5 years	46 males	34 females
Pseudotumor cerebri	[54]	1	8 years		Female
Meningoencephalitis	[55,56]	5	3.5 months-1 year	Males	Female
Pericardial effusion	[57]	440	Median age: 2 years	253 males	147 females
Valvular insufficiency	[58]	290	Median age: 2 years	155 males	135 females
Arrhythmia	[59]	1	1 year		Female
Prolonged PR interval	[60]	44	3 months-12 years	Males	Females
Peripheral gangrene	[61-63]	5	1 months-1 years	Males	Females
Aneurysms of various peripheral arteries	[64-66]	3	3 years-12 years	Males	
KD shock syndrome	[66]	103	1 months-18 years	Males	Females
KD shock syndrome	[67]	27	2 months-8.5 years	10 males	17 females
KD shock syndrome	[68]	5	5 months-11.5 years	4 males	1 female
KD with macrophage activation syndrome	[67-69]	10	4 months-12 years	Males	Female

## Conclusions

To conclude, KD diagnosis can be quite challenging for the pediatrician, since not all patients present with the classical symptoms and there are incomplete and atypical cases. Children presenting with fever lasting for more than five days without a clear cause of infection should raise the possibility of KD. In addition, one should keep in mind the various unusual clinical manifestations of KD to make a timely diagnosis and avoid complications such as CA aneurysms.
